# Supplementary material for: Release of CD36-associated cell-free mitochondrial DNA and RNA as a hallmark of space environment response
Source: Nat Commun. 2024 Jun 11;15:4814. doi: 10.1038/s41467-023-41995-z (PMC11166646; doi:10.1038/s41467-023-41995-z)
Supplement: Supplementary file 1 — Supplementary Information [file 41467_2023_41995_MOESM1_ESM.pdf]

Release of CD36-associated cell-free mitochondrial DNA and RNA as a hallmark of space environment response

Supplementary Figures

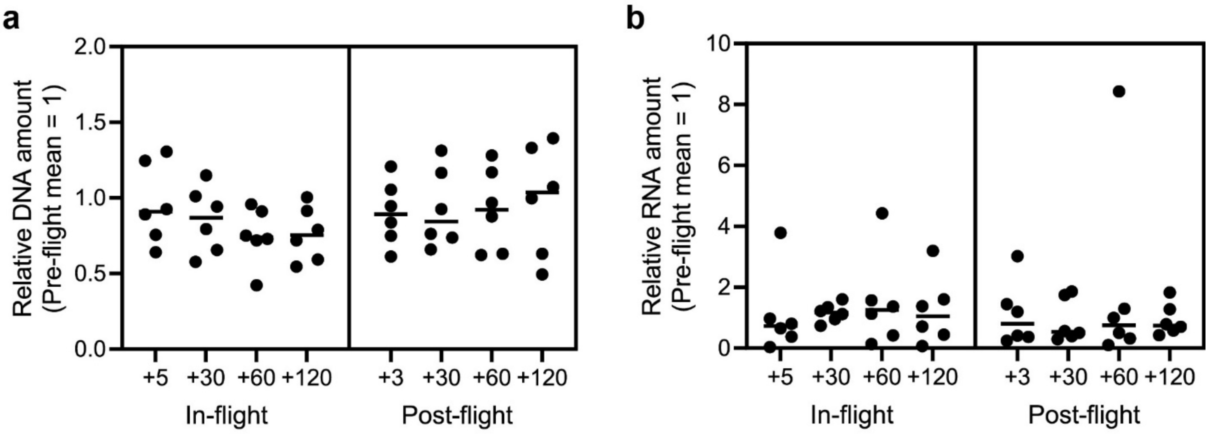

Supplementary Figure 1. Quantification of cfDNA and cfRNA.
(a) Relative amounts of DNA purified from plasma samples. DNA concentrations were measured by Qubit and values were divided by the mean of pre-flight samples for each astronaut.
(b) Relative RNA amounts.

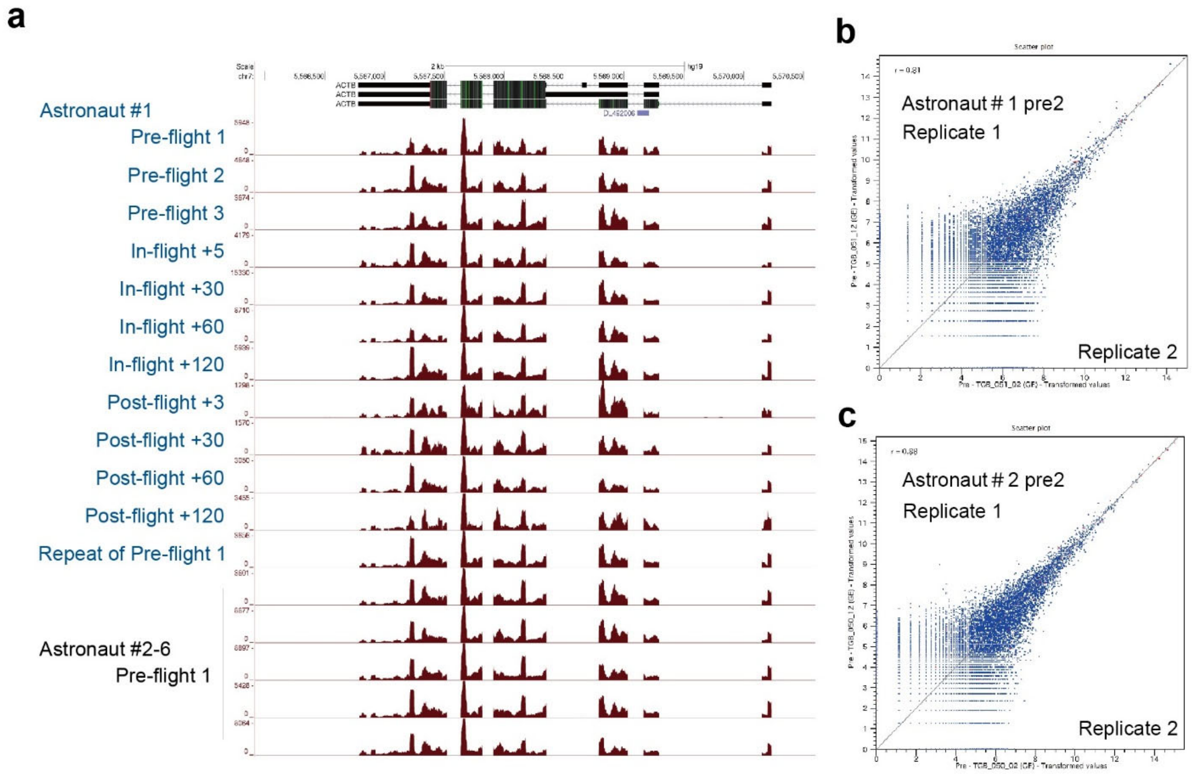

Supplementary Figure 2. Mapping and quantification of cfRNA-seq.
(a) Visualization of cfRNA-seq reads for all the samples from one astronaut and the first pre-flight time point samples from other astronauts in the UCSC Genome Browser.
(b, c) Quantification values from technical replicates for two astronaut samples as shown in scatter plots in Log2 scale.

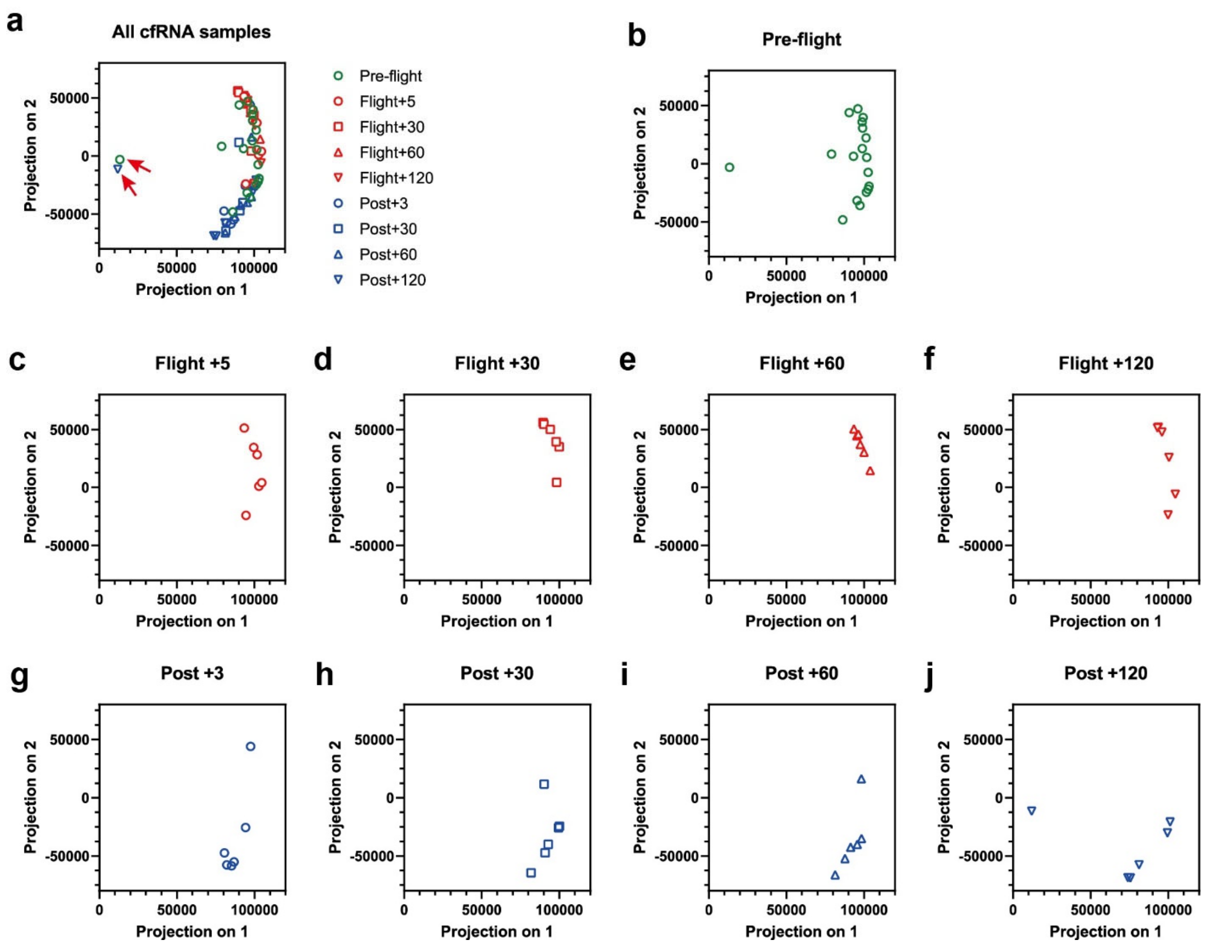

Supplementary Figure 3. PCA plots of human flight cfRNA-seq.
(a) PCA plot representing all the 66 samples normalized by the quantile method. The two outlier samples removed from subsequent analysis are indicated by red arrows.
(b-j) Panel (a) subdivided into each time point.

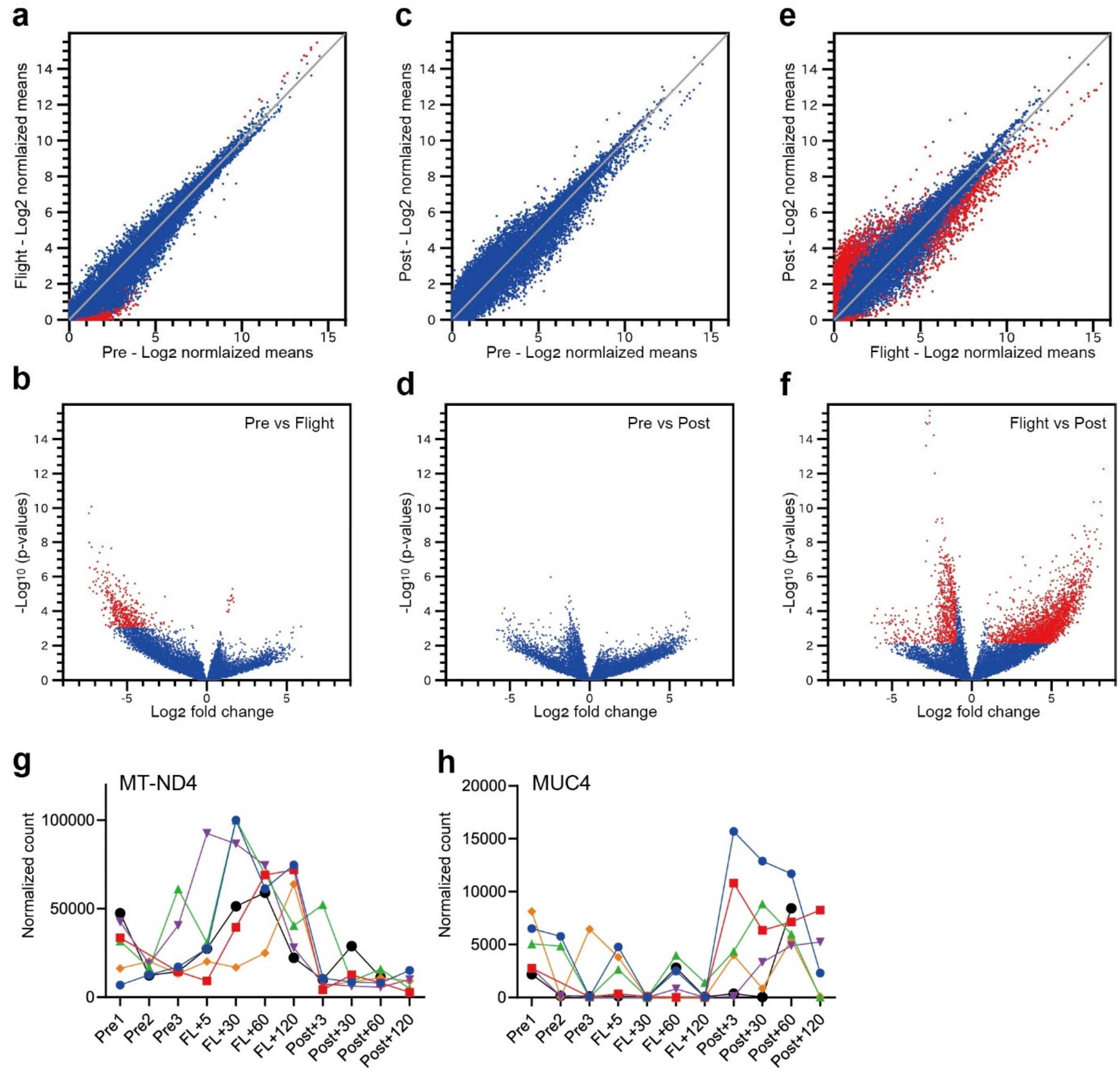

Supplementary Figure 4. Pairwise comparison of cfRNA-seq and variations between six astronauts across time course.

(a) Scatterplot of pre-flight and in-flight samples. Red dots represent the 472 genes with FDR-adjusted p-value < 0.05 and |fc| > 2.

(b) Volcano plot showing raw-p-value and fold change. Red dots represent the 472 filtered genes.

(c, d) Plots for pre-flight versus post-flight samples. Only one gene, marked as a red dot, met the filtering criteria.

(e, f) Plots for in-flight versus post-flight samples. Red dots represent the 3,574 genes that met the filtering criteria.

(g, h) Plots for the normalized counts of cfRNAseq results from the six astronauts. Variations between astronauts are represented by the 6 lines spanning the time course.

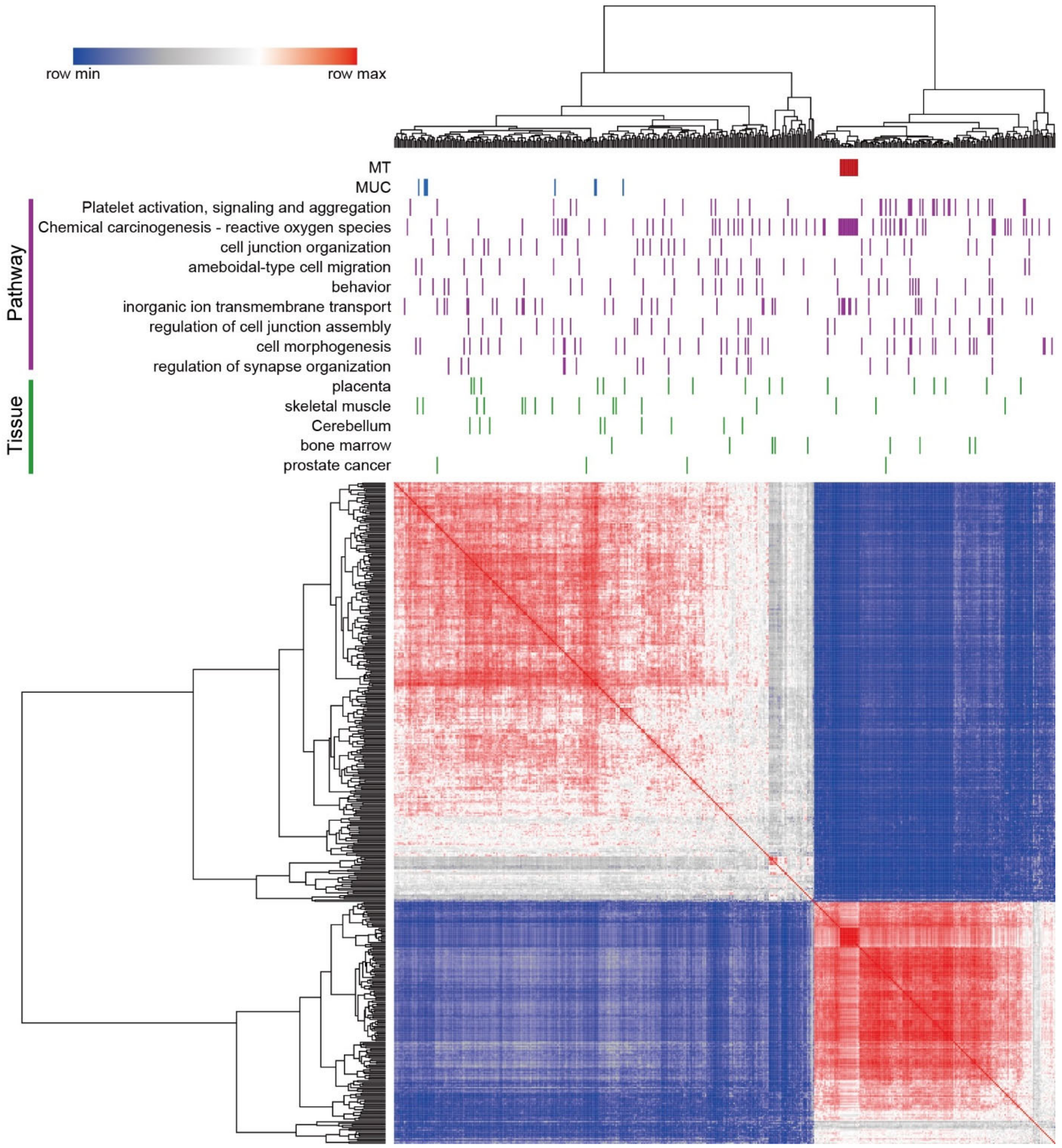

Supplementary Figure 5. Correlation analysis of differentially represented cfRNAs. Correlation analysis was performed between the 466 DRRs from Fig. 1. Normalized quantification values from the 64 samples (six astronauts, 11 time points, two samples removed) were used to estimate correlation values were displayed as a heatmap (minimum -0.74 in blue, maximum 1 in red). Gene annotations for mitochondrial genes (MT), mucin family genes, and genes from the Metascape pathway analysis in Fig. 2a, b are indicated. Note that cfRNAs for MT genes were clustered together.
