# Supplementary material for: Release of CD36-associated cell-free mitochondrial DNA and RNA as a hallmark of space environment response
Source: Nat Commun. 2024 Jun 11;15:4814. doi: 10.1038/s41467-023-41995-z (PMC11166646; doi:10.1038/s41467-023-41995-z)
Supplement: Supplementary file 3 — Description of Additional Supplementary Files [file 41467_2023_41995_MOESM3_ESM.pdf]

Description of Additional Supplementary Files

File Name: Supplementary Data 1
Description: **Pairwise comparison of plasma cfRNA-seq results.**
Pairwise comparisons were performed between pre-, in-, and post-flight cfRNA-seq quantification values. Total read counts were analyzed using the CLC Genomics Workbench's (CLC-GW, version 10.1.1) Empirical analysis of DGE function and genes were filtered by FDR-adjusted p-value < 0.05, |fold change| > 2. This identified 472 DRRs for pre- versus in-flight, one DRR for pre- versus post-flight, and 3,574 DRRs for in- versus post-flight comparisons, which were listed in three spread sheets. Each sheet contains gene names, p-values, fold change, FDR-adjusted p-values, and normalized mean counts.

File Name: Supplementary Data 2
Description: **466 DRRs from plasma cfRNA ANOVA filtering.**
The 466 DRRs shown in Fig. 1 listed with gene name; maximum difference between pre-, in-, and post-flight samples; fold change; p-values; FDR-adjusted p-values; and normalized mean values. Statistical analysis was performed using CLC-GW, Gaussian-based tests with ANOVA option.

File Name: Supplementary Data 3
Description: **Correlation matrix of plasma 466 cfRNA-seq DRRs and gene annotations.**
A correlation matrix for the 466 DRRs shown in Fig. 1 sorted by gene name. Genes are annotated with MT (mitochondrial genes), MUC (mucin genes), and Metascape pathway analysis terms.

File Name: Supplementary Data 4
Description: **Pairwise comparison of RNA-seq between input versus CD36 fraction.**
The 504 DRRs identified by a pairwise comparison of RNA-seq results between the input and the CD36 fraction. Total read counts were compared to identify DRRs (CLC GW, Empirical analysis of DGE, FDR-adjusted p-value < 0.05, |fold change| > 2). Shown are the gene name, p-values, fold change, FDR-adjusted p-values and normalized mean values of the three immunopurifications.

File Name: Supplementary Data 5
Description: **Tissue specificity analysis of RNAs enriched in CD36 fraction.**
Tissue analysis results from DAVID with the 406 DRRs enriched in the CD36 fraction compared to the input is shown. Tissue terms from the HPA database hits, gene counts, percentage of genes within annotated gene lists, p-values, gene names, fold enrichments, and FDR values from DAVID analysis output are shown.

File Name: Supplementary Data 6
Description: **Pairwise comparison of mouse plasma cfRNA-seq.**
The 467 DRRs from the pairwise comparison are shown with p-value and fold change. Normalized read counts and mean values are shown for artificial gravity (AG), microgravity (MG), and ground control (GC). Statistical analysis was performed using CLC-GW, Empirical analysis of DGE function.
